# Blood serotonin, ionized magnesium, cardiac troponin I, N-terminal pro-brain natriuretic peptide concentrations, and mitral valve billowing in horses with mitral valve regurgitation

**DOI:** 10.1093/jvimsj/aalag280

**Published:** 2026-11-06

**Authors:** Marie Demeyere, Alexander Dufourni, Lize-Maria Verhaeghe, Ellen Paulussen, Lennert Heirman, Siegrid De Baere, Siska Croubels, Tine De Backer, Gunther van Loon, Annelies Decloedt

**Affiliations:** Equine Cardioteam Ghent, Department of Internal Medicine, Reproduction and Population Medicine, Faculty of Veterinary Medicine, Ghent University, Merelbeke-Melle, Belgium; Equine Cardioteam Ghent, Department of Internal Medicine, Reproduction and Population Medicine, Faculty of Veterinary Medicine, Ghent University, Merelbeke-Melle, Belgium; Equine Cardioteam Ghent, Department of Internal Medicine, Reproduction and Population Medicine, Faculty of Veterinary Medicine, Ghent University, Merelbeke-Melle, Belgium; Equine Cardioteam Ghent, Department of Internal Medicine, Reproduction and Population Medicine, Faculty of Veterinary Medicine, Ghent University, Merelbeke-Melle, Belgium; Equine Cardioteam Ghent, Department of Internal Medicine, Reproduction and Population Medicine, Faculty of Veterinary Medicine, Ghent University, Merelbeke-Melle, Belgium; Laboratory of Pharmacology and Toxicology, Department of Pathobiology, Pharmacology and Zoological Medicine, Faculty of Veterinary Medicine, Ghent University, Merelbeke-Melle, Belgium; Laboratory of Pharmacology and Toxicology, Department of Pathobiology, Pharmacology and Zoological Medicine, Faculty of Veterinary Medicine, Ghent University, Merelbeke-Melle, Belgium; Ghent University Hospital, Department of Cardiology, Ghent University, Ghent, Belgium; Equine Cardioteam Ghent, Department of Internal Medicine, Reproduction and Population Medicine, Faculty of Veterinary Medicine, Ghent University, Merelbeke-Melle, Belgium; Equine Cardioteam Ghent, Department of Internal Medicine, Reproduction and Population Medicine, Faculty of Veterinary Medicine, Ghent University, Merelbeke-Melle, Belgium

**Keywords:** biomarkers, echocardiography, equine cardiology, mitral valve insufficiency, mitral valve prolapse, platelet-poor plasma

## Abstract

**Background:**

Serotonin, ionized magnesium (iMg), cardiac biomarkers, and mitral valve prolapse are extensively investigated in humans and dogs with mitral valve regurgitation (MR), but not in horses.

**Hypothesis/Objectives:**

To investigate serotonin, iMg, cardiac troponin I (cTnI), and N-terminal pro-brain natriuretic peptide (NT-proBNP) concentrations and degree of mitral valve billowing (MVB) in horses with MR.

**Animals:**

Eighty controls (no/trivial MR), 122 horses with MR (92 mild, 24 moderate, 6 severe).

**Methods:**

Prospective cross-sectional observational study. All horses underwent echocardiography with MVB distance assessment and MR classification. Platelet-poor plasma serotonin and serum iMg, cTnI, and NT-proBNP concentrations were determined. Controls and horses with different MR grades, biomarker concentrations, and MVB distance were compared using (non-)parametric tests.

**Results:**

Ionized magnesium concentration was lower in MR compared to controls (mean difference, 0.03 mmol/L; 95% CI, 0.01-0.05; *P* = .01), but showed no correlation with billowing distance. The billowing distance was higher in horses with MR than in controls (septal: median difference, 0.2 cm; 95% CI, 0.1-0.3; *P* < .001; parietal: median difference, 0.3 cm; 95% CI, 0.2-0.3; *P* < .001), and in horses with a crescendo compared to a holosystolic MR jet (septal: median difference, 0.2 cm; 95% CI, 0.1-0.3; *P* = .003; parietal: median difference, 0.1 cm; 95% CI, 0.0-0.2; *P* = .01). No differences in serotonin, cTnI, and NT-proBNP concentrations were detected.

**Conclusions and clinical importance:**

Mitral valve billowing is to some extent likely physiological. The association of serotonin and iMg in MVB and MR requires further investigation. Both cTnI and NT-proBNP showed limited use as biomarkers of MR severity.

**Abstract (Dutch):**

*Achtergrondinformatie:* Serotonine, geïoniseerd magnesium (iMg), cardiale biomarkers en mitraalklep prolaps werden reeds uitgebreid onderzocht bij mensen en honden met mitraalklep regurgitatie (MR), in tegenstelling tot paarden met MR.

*Hypothese/Doelstelling:* Het onderzoeken van de serotonine, iMg, cardiaal troponine I (cTnI) en N-terminal pro-brain natriuretic peptide (NT-proBNP) concentraties en de mate van bolling van de mitraalklep naar het linker atrium (MVB) bij paarden met MR.

*Studiepopulatie:* Tachtig controle paarden (geen/triviaal MR), 122 paarden met MR (92 mild, 24 matig, 6 erg).

*Materiaal en methoden:* Prospectieve cross-sectionele observationele studie. Alle paarden ondergingen echocardiografie, waarna de mate van MVB gemeten werd en MR classificatie uitgevoerd werd. De concentraties van serotonine in bloedplaatjesarm plasma en van iMg, cTnI en NT-proBNP in serum werden bepaald. Controle paarden en paarden met verschillende MR graden, biomarker concentraties en de mate van MVB werden onderling vergeleken aan de hand van (non-)parametrische testen.

*Resultaten:* De concentratie iMg was lager bij paarden met MR in vergelijking met controle paarden (gemiddeld verschil, 0.03 mmol/L; 95% BI, 0.01-0.05; *P* = 0.01), maar vertoonde geen correlatie met de mate van MVB. De mate van MVB was hoger bij paarden met MR dan bij controle paarden (septaal: mediaan verschil, 0.2 cm; 95% BI, 0.1-0.3; *P* < 0.001; pariëtaal: mediaan verschil, 0.3 cm; 95% BI, 0.2-0.3; *P* < 0.001), en bij paarden met een crescendo MR jet in vergelijking met paarden met een holosystolische MR jet (septaal: mediaan verschil, 0.2 cm; 95% BI, 0.1-0.3; *P* = 0.003; pariëtaal: mediaan verschil, 0.1 cm; 95% BI, 0.0-0.2; *P* = 0.01). Er werden geen verschillen gedetecteerd in de concentraties van serotonine, cTnI en NT-proBNP.

*Conclusies en klinisch belang:* De mate van MVB is tot op een bepaalde hoogte waarschijnlijk fysiologisch. De rol van serotonine en iMg in MVB en MR vergt verder onderzoek. Zowel cTnI als NT-proBNP vertoonden een beperkte meerwaarde als biomarkers bij het bepalen van de ernst van MR.

## Introduction

While the pathophysiology of mitral valve regurgitation (MR) remains largely unknown in horses, molecular mechanisms involving 5-hydroxytryptamine or serotonin (5-HT) activating valve interstitial cells (VICs) are suggested as contributing factors to myxomatous mitral valve disease (MMVD) in humans and dogs.^1^ Although activation of the 5-HT pathway and VICs remains incompletely understood, high blood 5-HT concentrations are reported in humans with MR,^1–7^ whereas studies in dogs with MMVD report conflicting results.^8–10^ Additionally, mitral valve prolapse (MVP) is a well-defined cardiac disease in humans and dogs. Hypomagnesemia is associated with MVP resulting in MR,^11^^,^^12^ potentially through left ventricular (LV) dyssynchrony and impaired collagen metabolism.^13^ Magnesium supplementation in MVP patients and MMVD dogs has beneficial effects.^11^^,^^14^^,^^15^ Given the absence of established equine-specific diagnostic MVP criteria and different morphology of the equine mitral valve, the degree of systolic displacement of the mitral valve leaflet(s) above the mitral annular plane on a right parasternal four chamber view was descriptively termed mitral valve billowing (MVB). No implication regarding physiological or pathological status is intended by this terminology. In horses, 5-HT and magnesium have been studied in various diseases,^16^^,^^17^ but data on their role in MR and MVB are lacking.

Beyond elucidating MR pathophysiology, identifying cardiac biomarkers associated with its hemodynamic consequences is important, as they might allow detection of chamber remodeling before such changes become apparent on echocardiography.^18^ N-terminal pro-brain natriuretic peptide (NT-proBNP) is the biomarker of choice in both human and canine cardiology, given its well-established clinical utility as an early marker of cardiac enlargement.^19–21^ Elevated NT-proBNP concentrations are directly correlated with higher New York Heart Association functional classes in primary MR in humans^22^ and with advanced MMVD stages in dogs.^23^^,^^24^ Cardiac troponin I (cTnI) is released into the circulation after myocardial injury, which in MR can result from myocardial stretch caused by volume overload, potentially leading to higher intracardiac pressures and chamber remodeling.^25^ In humans and dogs with mitral valve disease, higher cTnI concentrations are associated with shorter survival times and increased risk of cardiac-related death.^26^^,^^27^ In horses, cTnI and NT-proBNP concentrations have only been sparsely investigated.^28–30^

This study aimed to investigate ionized magnesium (iMg), 5-HT, NT-proBNP, and cTnI concentrations in healthy horses and horses with MR, and to evaluate the association between iMg concentration and MVB distance. We hypothesized that increased MR severity would be associated with elevated 5-HT, NT-proBNP, and cTnI and decreased iMg concentrations, and that hypomagnesemia would be correlated with MVB distance.

## Materials and methods

### Animals and study design

This prospective cross-sectional observational study contained client-owned horses, university-owned horses and horses donated for scientific research with no or trivial MR (healthy group) or mild, moderate, or severe MR (MR group) that underwent a cardiac examination. The inclusion criteria were: Warmblood, Trotter, or Thoroughbred breed, ≥2 years of age, body weight 400-700 kg, absence of congenital cardiac abnormalities, good-quality echocardiographic images, and no concurrent valvular regurgitation exceeding the severity of the MR classification.

### Echocardiography: assessment of mitral valve regurgitation and billowing

An echocardiographic examination was performed, including two-dimensional and color flow Doppler images from a right and left parasternal view.^31^ Mitral valve regurgitation was classified according to a previously described scoring system^32^ including left atrial (LA) area measurement, subjective assessment of LA size, and regurgitant jet size and duration (Supplementary item A). The degree of MVB was quantified as the systolic displacement of the mitral valve leaflet(s) above the mitral annular plane on a right parasternal four chamber view. First, the mitral annulus diameter was measured by connecting the ventricular side of the mitral annular hinge points. Subsequently, MVB distance was measured from the ventricular side of the mitral annulus to the atrial side of the most billowing portion of the septal and parietal leaflet extending into the LA^33^^,^^34^ (Figure 1).

**Figure 1:**
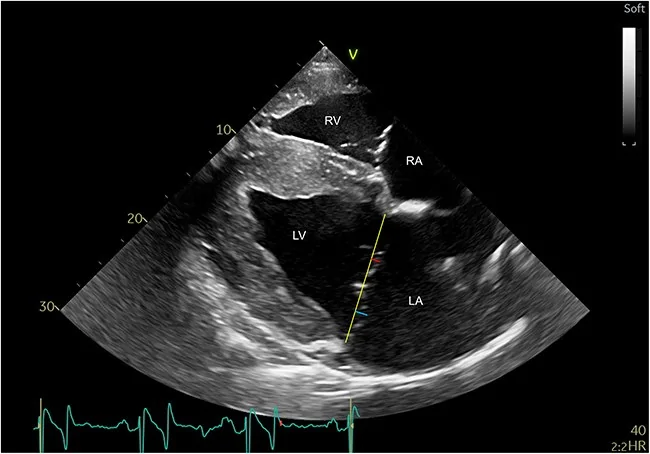
Mitral valve billowing measurement at end-systole, one frame before mitral valve opening, on a right parasternal four chamber view in horses. Yellow line: mitral annulus diameter, red line: billowing distance of the septal leaflet of the mitral valve, blue line: billowing distance of the parietal leaflet of the mitral valve. Abbreviations: LA = left atrium; LV = left ventricle; RA = right atrium; RV = right ventricle.

### Sample collection and analysis

Blood was collected in serum tubes and cooled ethylenediaminetetraacetic acid tubes containing stabilizing products to prevent in vitro platelet activation. Platelet-poor plasma for 5-HT concentration analysis was obtained following a previously described protocol.^35^ Serum was used for determining iMg, cTnI, and NT-proBNP concentrations. When cTnI concentration was >0.06 ng/mL, additional cTnI and cTnT tests were performed to check for false elevations. Samples were stored at −20 or −80 °C aiming for a maximal storage time of 12 months.

### Statistical analysis

Data were tested for normality. Normally distributed data are reported as mean ± standard deviation and non-normally distributed data as median, interquartile range [Q1; Q3], and range (minimum-maximum). Demographic, echocardiographic, and biomarker data were compared between four groups (healthy, mild, moderate, and severe MR) and between two groups (healthy and MR) using (non-)parametric tests. Multiple linear regression determined the effect of MR and analysis batch on biomarker concentration. Spearman’s rho assessed correlations between biomarkers and echocardiographic data (cardiac dimensions and billowing distance). *P*-value <.05 was considered significant.

More detailed description of materials and methods can be found in the Appendix.

## Results

The study group consisted of 202 horses. Demographic characteristics of the study cohort are summarized in Table 1. The healthy group (no or trivial valvular regurgitations) consisted of 80 horses, while 122 horses were in the MR group (92 mild, 24 moderate, 6 severe). Two horses were diagnosed with atrial fibrillation and congestive heart failure due to primary moderate or severe mitral valve regurgitation.

**Table 1:** Demographic, echocardiographic and biomarker (5-HT, iMg, NT-proBNP, and cTnI) data in healthy horses and horses with mild, moderate, and severe mitral valve regurgitation.

	Healthy/Trivial MR (*n* = 80)	Mild MR (*n* = 92)	Moderate MR (*n* = 24)	Severe MR (*n* = 6)	Total MR (*n* = 122)
**Demographic data**					
**Breed (count)**					
** Warmblood Trotter Thoroughbred**	76 2 2	86 1 5	24 0 0	6 0 0	116 1 5
**Sex (count)**					
** Mare Gelding Stallion**	35 39 6	32 57 3	1 21 2	2 4 0	35 82 5
**Age (years)**	8 [5-15] (2-31)	8 [5-11] (2-26)	7 [5-8] (3-26)	15 [7-20] (5-30)	8 [5-12] (2-30)
**Height (cm)**	166 [162-170] (150-188)^a^	170 [165-175] (160-183)^b^	170 [165-172] (160-175)^a,b^	167 [164-175] (160-180)^a,b^	170 [165-174] (160-183)^*^
**Body weight (kg)**	550 ± 61	557 ± 54	548 ± 57	528 ± 78	554 ± 56
**Echocardiographic data**					
**LAA_s,end_[500] (cm^2^)**	79.4 ± 9.5^a^	89.4 ± 8.7^b^	106.9 ± 10.6^c^	150.2 ± 28.5^d^	95.8 ± 17.8^*^
**Subjective LA size and shape (count) Normal Mild enlargement Moderate enlargement Severe enlargement**	80 (100%) 0 (0%) 0 (0%) 0 (0%)	65 (71%) 27 (29%) 0 (0%) 0 (0%)	6 (25%) 17 (71%) 1 (4%) 0 (0%)	0 (0%) 1 (17%) 3 (50%) 2 (33%)	71 (58%) 45 (37%) 4 (3%) 2 (2%)
**Regurgitant jet size (count) No jet or jet < 1/5 of LA area Jet ≤ 1/3 of LA area Jet ≤ 1/2 of LA area Jet > 1/2 of LA area**	66 (83%) 14 (17%) 0 (0%) 0 (0%)	2 (2%) 51 (55%) 33 (36%) 6 (7%)	0 (0%) 3 (12%) 9 (38%) 12 (50%)	0 (0%) 0 (0%) 1 (17%) 5 (83%)	2 (2%) 54 (44%) 43 (35%) 23 (19%)
**Jet duration (if present; count) Crescendo jet Holosystolic jet**	15 (19%) 25 (31%)	35 (38%) 57 (62%)	4 (17%) 20 (83%)	0 (0%) 6 (100%)	39 (32%) 83 (68%)
**Mitral annulus diameter [500] (cm)**	9.0 ± 0.7^a^	9.6 ± 0.7^b^	10.2 ± 0.6^c^	11.5 ± 0.7^d^	9.8 ± 0.8^*^
**MVB distance septal leaflet (cm)**	0.6 [0.5-0.8] (0.2-1.3)^a^	0.8 [0.6-1.0] (0.3-1.4)^b^	0.9 [0.7-1.3] (0.3-1.7)^b^	0.8 [0.6-1.3] (0.6-1.4)^a,b^	0.8 [0.6-1.0] (0.3-1.7)^*^
**MVB distance parietal leaflet (cm)**	0.4 [0.4-0.7] (0.1-1.1)^a^	0.7 [0.6-0.9] (0.3-1.3)^b^	0.9 [0.7-0.9] (0.4-1.5)^b^	0.8 [0.7-1.4] (0.7-1.5)^b^	0.7 [0.6-0.9] (0.3-1.5)^*^
**Biomarker data**					
**5-HT concentration (ng/mL)**	6.00 [2.84-9.17] (0.71-66.25)	5.30 [3.37-11.41] (0.96-109.50)	4.55 [2.80-8.52] (1.06-133.17)	14.64 [5.05-44.25] (3.92-52.06)	5.30 [3.29-11.41] (0.96-133.17)
	(*n* = 76)	(*n* = 81)	(*n* = 20)	(*n* = 4)	(*n* = 105)
**5-HT storage time (days)**	111 [58-143] (11-214)	87 [51-120] (12-228)	107 [71-165] (22-225)	55 [43-127] (39-150)	91 [54-123] (12-228)
**iMg concentration (mmol/L)**	0.43 ± 0.08	0.40 ± 0.08	0.38 ± 0.08	0.39 ± 0.08	0.40 ± 0.08^*^
	(*n* = 80)	(*n* = 92)	(*n* = 24)	(*n* = 6)	(*n* = 122)
**iMg storage time (days)**	81 [56-278] (2-510)	75 [31-213] (1-421)	153 [62-306] (9-440)	81 [37-340] (28-440)	81 [40-254] (1-440)
**NT-proBNP concentration (pg/mL)**	31.2 [31.2-33.4] (31.2-134.1) (n = 80)	31.2 [31.2-65.4] (31.2-147.2) (n = 92)	31.2 [31.2-47.1] (31.2-148.0) (*n* = 23)	31.2 [31.2-55.3] (31.2-67.6) (*n* = 6)	31.2 [31.2-64.3] (31.2-148.0)^*^
**NT-proBNP storage time (days)**	168 [112-189] (3-490)^a^	119 [69-175] (6-322)^b^	107 [71-200] (28-420)^a,b^	79 [31-188] (20-222)^a,b^	118 [70-176] (6-420)^*^
**cTnI concentration (ng/mL)**	0.01 [0.01-0.03] (0.01-1.37)	0.01 [0.01-0.02] (0.01-0.54)	0.01 [0.01-0.01] (0.01-0.49)	0.02 [0.01-0.29] (0.01-1.01)	0.01 [0.01-0.02] (0.01-1.01)
	(*n* = 79)	(*n* = 92)	(*n* = 23)	(*n* = 6)	(*n* = 121)
**cTnI storage time (days)**	100 [73-135] (9-356)^a,b^	82 [31-132] (7-267)^a^	135 [95-161] (5-286)^b^	140 [89-205] (35-286)^a,b^	98 [37-147] (5-286)

Abbreviations: 5-HT = serotonin; cTnI = cardiac troponin I; iMg = ionized magnesium; LA = left atrial; LAA_s,end_[500] = left atrial area at end-systole, normalized to a bodyweight of 500 kg using allometric scaling; MVB = mitral valve billowing; MR = mitral valve regurgitation; NT-proBNP = N-terminal pro-brain natriuretic peptide.

Normally distributed variables are reported as mean ± standard deviation and non-normally distributed variables are reported as median, interquartile range [Q1; Q3], and range (minimum-maximum). Information regarding height at the withers was missing in 3 horses (2 healthy and 1 mild MR). A crescendo jet was defined as < 1/5 of LA area during the first half of systole, but ≥1/5 of LA area in the second half of systole. Data regarding mitral annulus diameter and MVB distance measurements were missing in 16 horses (11 healthy, 2 mild MR, 1 moderate MR, and 2 severe MR). An asterisk (^*^) indicates a significant difference between the MR group and the healthy group. Different superscripts (a-d) indicate a significant difference between subgroups (healthy, mild MR, moderate MR and severe MR). A *P*-value <.05 was considered statistically significant.

In 16 horses (11 healthy, 2 mild MR, 1 moderate MR, and 2 severe MR), quantification of MVB was not feasible due to drop-out at the leaflet level or acoustic shadowing from the lungs obscuring the mitral valve during systole. The mitral annulus diameter and LA area increased significantly with increasing MR severity (*P* ≤ .004; Table 1). The billowing distance measured for both the septal and parietal leaflet of the mitral valve was significantly higher in horses with MR compared to horses without MR (septal: median difference, 0.2 cm; 95% CI, 0.1-0.3; *P* < .001; parietal: median difference, 0.3 cm; 95% CI, 0.2-0.3; *P* < .001). No significant differences were observed between MR subgroups (*P* > .05; Table 1). In case of a subjectively assessed crescendo MR jet, the MVB distance was higher for both the septal and parietal mitral valve leaflet compared to a holosystolic MR jet (septal: median difference, 0.2 cm; 95% CI, 0.1-0.3; *P* = .003; parietal: median difference, 0.1 cm; 95% CI, 0.0-0.2; *P* = .01).

### Ionized magnesium

The iMg concentration in serum was measured in all 202 horses. The proportion of horses with hypomagnesemia (<0.43 mmol/L)^36^ was 50% and 64% in the healthy and MR group, respectively (Cramer’s V = 0.138, *P* = .049). The MR group showed a significantly lower iMg concentration compared to the healthy group (mean difference, 0.03 mmol/L; 95% CI, 0.01-0.05; *P* = .01). When the four MR severity groups were compared, the difference was not statistically significant (*P* = .05; Table 1). Ionized magnesium concentration did not differ between horses with a crescendo MR jet and with a holosystolic MR jet (*P* = .93). No significant differences in storage time were detected when either two (*P* = .26) or four (*P* = .10) groups were compared (Table 1). Serum iMg concentration did not correlate with mitral annulus diameter or billowing distance of the septal and parietal leaflet (*P* = .53, *P* = .96, and *P* = .45, respectively).

### Serotonin

Platelet-poor plasma 5-HT concentration was analyzed in 181 out of 202 horses and revealed no correlation with MVB (septal: rs = −0.02, *P* = .76; parietal: rs = 0.05, *P* = .54). Serotonin concentration did not differ between the healthy and MR group (*P* = .66) or between healthy horses, mild MR, moderate MR, and severe MR (*P* = .36; Table 1). The median storage time was 98 days ([54-142] days, (11-228) days), with no significant differences in storage duration between groups (*P* > .05; Table 1).

### N-terminal pro-brain natriuretic peptide

The NT-proBNP concentration was measured in 201 out of 202 horses. Serum NT-proBNP concentration was significantly higher in horses with MR (31.2 pg/mL ([31.2-64.3] pg/mL, (31.2-148.0) pg/mL)) compared to healthy horses (31.2 pg/mL ([31.2-33.4] pg/mL, (31.2-134.1) pg/mL); *P* = .01), although the median value was the same in both groups, which explains the median difference of 0.0 pg/mL (Table 1). There were no significant differences in NT-proBNP concentration between healthy horses and horses with mild, moderate, and severe MR (*P* = .07; Table 1). The LA area at end-systole measured for MR classification showed a weak correlation with the NT-proBNP concentration (rs = 0.16, *P* = .02), while the subjective assessment of LA size and shape was not associated (rs = 0.04, *P* = .61). Billowing distance of the septal leaflet was not correlated with the NT-proBNP concentration (rs = −0.01, *P* = .99), whereas the parietal leaflet showed a weak correlation (rs = 0.18, *P* = .01).

The median time between sample collection and analysis was 144 days ([82-185] days, (3-490) days). Storage duration was longer in healthy horses (168 days ([112-189] days, (3-490) days)) compared to horses with MR (118 days ([70-176] days, (6-420) days); *P* = .01; Table 1). The NT-proBNP concentration was significantly higher in the fourth (93.1 pg/mL ([72.3-118.3] pg/mL, (47.1-148.0) pg/mL)) and fifth (68.2 pg/mL ([44.2-83.8] pg/mL, (31.2-117.4) pg/mL)) analysis batch compared to the first (31.2 pg/mL ([31.2-31.2] pg/mL, (31.2-58.3) pg/mL); *P* < .001), second (31.2 pg/mL ([31.2-31.2] pg/mL, (31.2-36.7) pg/mL); *P* < .001) and third (31.2 pg/mL ([31.2-32.3] pg/mL, (31.2-73.2) pg/mL); *P* < .001) batch. After multiple linear regression analysis, the observed difference in NT-proBNP concentration between the healthy group and the MR group was due to the different analysis batches (*P* < .001) rather than due to the presence or absence of MR (*P* = .26).

### Cardiac troponin I

The concentration of cTnI was determined in serum in 200 out of 202 horses. The median time between sample collection and analysis was 100 days ([51-140] days, (5-356) days). Serum cTnI concentration was not correlated with MVB (septal: rs = 0.08, *P* = .26; parietal: rs = 0.11, *P* = .15). No significant differences were found either between the healthy and MR groups (*P* = .14) or among the four groups (*P* = .08; Table 1). In 15 out of 200 horses (9 healthy, 3 mild MR, 2 moderate MR, and 1 severe MR) a cTnI concentration > 0.06 ng/mL was found (0.13 ng/mL ([0.09-0.49] ng/mL, (0.07-1.37) ng/mL)). The STAT cTnI test revealed an elevated cTnI concentration (>0.06 ng/mL) in 4 out of 15 horses (2 healthy, 1 moderate MR, and 1 severe MR), with a median value of 0.17 ng/mL ([0.08-0.26] ng/mL, (0.07-0.27) ng/mL). Cardiac troponin T concentration was increased (>14 pg/mL) in the same 4 out of 15 horses, with a median value of 32.85 pg/mL ([19.00-52.70] pg/mL, (17.60-56.10) pg/mL).

Spearman’s rho correlation coefficient did not reveal any correlation between cTnI and NT-proBNP concentration in equine serum samples (rs = 0.02, *P* = .74).

## Discussion

The main findings of this study are a statistically significant lower iMg concentration in horses with MR compared to healthy horses, a higher MVB distance in horses with MR, and a higher MVB distance in horses with a crescendo MR jet compared to a holosystolic MR jet. Ionized magnesium concentration was not correlated with MVB distance and no clinically relevant differences in 5-HT, NT-proBNP, and cTnI concentrations were found between healthy horses and horses with MR.

In human cardiology, MVP is defined as a systolic displacement of one or both mitral valve leaflets ≥2 mm above the mitral annular plane, which occurs in 2%-3% of the general population.^37^ The prolapse distance is measured as the maximum excursion of both mitral leaflets beyond a line connecting the mitral annular hinge points.^38^ In canine cardiology, MVP severity is more commonly assessed subjectively as mild, moderate, or severe, relative to a line connecting the mitral annular hinge points.^39^ In the present study, MVB was defined as the degree of systolic displacement of one or both mitral valve leaflets above the mitral annular plane on a right parasternal four chamber view, without specification of a distinct cutoff above which MVB should be considered abnormal or classified as MVP. Therefore, to some degree, MVB is likely a physiological finding in horses. When discussing MVB in horses, it is important to consider the mitral valve anatomy in the horse and the measurement method used. Compared to humans and dogs, the implantation of the mitral valve differs in horses resulting in a distinct valve position at end-systole. In humans and dogs, the mitral annulus and valve are saddle-shaped at end-systole, meaning that the coaptation point of the mitral valve leaflets is located at the ventricular side of a line connecting the mitral annular hinge points on a parasternal long-axis view of the left ventricle.^40^^,^^41^ Even though not strictly defined, measurement at the mitral annular hinge point, as a reference for MVP assessment, is mostly depicted at the ventricular side of the mitral valve in both human and canine cardiology.^42–44^ Displacement of the leaflets ≥2 mm above this mitral annular plane indicates a substantial change from normal and might be related to marked annular dilatation, papillary muscle fibrosis, or both. In normal horses, however, at end-systole the septal and parietal mitral valve leaflet form an almost straight line at the level of the mitral annular plane on a right parasternal four chamber view, connecting the mitral annular hinge points, seemingly inconsistent with the saddle-shaped mitral annulus and valve in humans and dogs. Furthermore, the equine mitral valve is thicker compared to the healthy human and canine mitral valve and is included in the MVB distance due to our measurement method. This explains why the minimal MVB distance measured was never zero or a negative value, but was 0.2 cm for the septal leaflet and 0.1 cm for the parietal leaflet. We suggest that MVB in horses exists along a continuum ranging from absence of MVB to a distinct threshold of MVB that has not yet been defined, beyond which the condition could be considered clinically relevant. While a leaflet displacement of ≥2 mm above the mitral annular plane is considered MVP in the small human heart, this threshold likely requires adjustment when applied to the much larger equine heart. Further investigation in horses is required to obtain a better understanding of the mitral annulus and valve shape and dynamics throughout the cardiac cycle in order to define what degree of MVB is considered physiologic and whether excessive MVB should be considered pathologic. At present, data regarding MVB in horses is scarce. One study reported subjective MVP in 26% of horses with severe MR, using displacement of a mitral valve leaflet beyond the mitral annular plane as reference, even though there is no commonly accepted definition of MVP in horses available.^45^ More recent research tried to quantify MVB distance, however, equine-specific cutoffs need to be defined to distinguish normal MVB from pathological MVP.^33^^,^^34^

Even though mostly benign in humans,^46^ a subgroup of MVP patients is at risk for ventricular arrhythmias and sudden cardiac death,^47^ potentially related to papillary muscle fibrosis.^38^ The higher billowing distance for both mitral valve leaflets detected in horses with a subjectively assessed crescendo MR jet compared to a holosystolic MR jet, might suggest that the papillary muscles play a role in equine MVB. A hypothetical mechanism could be reduction in papillary muscle contraction strength towards the end of systole, potentially due to magnesium disorders or fibrosis, reducing chordal tension and allowing displacement of the mitral valve leaflets towards the LA, resulting in MVB and a crescendo MR jet.^48^ An association between MVP and MR exists in Cavalier King Charles Spaniels, in which the degree of prolapse is correlated with MR severity and MMVD progression.^49^^,^^50^ However, the increased MVB in horses with MR might reflect adaptive remodeling of the mitral valve and left-sided chambers secondary to MR, rather than serving as its primary cause.

In human cardiology, an association between hypomagnesemia and MVP is suggested, with hypomagnesemia present in 60%-81% of MVP patients.^11^^,^^14^ Hypomagnesemia is reported in dogs with MMVD and MVP, but prevalences vary across studies.^12^^,^^51^^,^^52^ Pedersen and Mow did not find a correlation between both MR jet size and MVP severity and plasma magnesium concentration,^52^ which seems to be similar to our findings. However, direct comparison is difficult as plasma magnesium and serum iMg represent different magnesium fractions and might vary independently. Hypomagnesemia was a frequent finding in the group of healthy horses. Although data on iMg concentrations in healthy horses are limited,^17^ the values observed were in the lower range of reported values.^53^^,^^54^ Sample storage time might have contributed to lower iMg concentrations through pH-related changes, although this effect is considered limited in horses.^17^^,^^55^

In humans, various hypotheses linking hypomagnesemia and MVP have been put forward. A potential mechanism involves stress-induced adrenergic activity, which stimulates the renin-angiotensin-aldosterone system leading to renal magnesium loss.^56^ In human patients, hypomagnesemia might contribute to functional MVP by inducing LV contractile dyssynchrony and to mitral valve degeneration through altered collagen synthesis.^13^ After magnesium supplementation, several studies in human cardiology report a decrease in MR severity and LA size as well as an improvement in symptoms related to MVP, e.g., anxiety, chest pain, and weakness.^11^^,^^14^ However, these symptoms can also be related to the previously mentioned hyperadrenergic activity,^57^ resulting in an increased blood pressure and heart rate. Relief of symptoms and decrease in MR severity and LA size could thus be merely due to the anti-adrenergic and vasodilatory effect of magnesium, decreasing blood pressure and heart rate.^11^ In dogs, a diet including magnesium and other cardioprotective nutrients slows or prevents the progression of early MMVD.^15^

Studies investigating circulating 5-HT concentrations in canine MMVD report inconsistent findings, with some studies describing associations with disease severity or echocardiographic variables, while others did not.^8–10^^,^^58^^,^^59^ Similar to our findings, Reimann et al. found no significant differences in 5-HT concentrations among MMVD stages in Cavalier King Charles Spaniels. They also reported a pattern comparable to that observed in the present study, with a gradual decrease in 5-HT concentrations in stage B1/mild MR and stage B2/moderate MR and increased 5-HT concentrations in stage C/severe MR.^10^ The lack of differences in 5-HT concentrations between MR groups in our study suggests that circulating 5-HT concentrations have limited value as a marker of MR severity or cardiac remodeling in horses. Nevertheless, 5-HT molecular mechanisms are still considered as one of the key factors in the pathophysiology of human and canine MMVD.^1–6^ Proposed mechanisms are 5-HT receptor overexpression, 5-HT reuptake transporter downregulation, and local autocrine valvular 5-HT production,^2–5^^,^^60^ which promote VICs activation and extracellular matrix remodeling, resulting in thickening and degeneration of the mitral valve.^61^ The systemic 5-HT concentration investigated in our study might thus represent only one component of a broader pathophysiological framework.

Comparison of reported 5-HT concentrations across studies should be interpreted with caution because both sample type and analytical method can substantially influence measured values. Serum typically contains higher 5-HT concentrations than plasma due to release of platelet-stored 5-HT during clot formation.^62^ Furthermore, plasma 5-HT concentrations differ between platelet-rich and platelet-poor plasma, as platelet activation in platelet-rich plasma, due to, e.g., sample handling, endogenous plasma thrombin, and temperature shift,^63–65^ could artifactually increase measured concentrations.^8^ Therefore, platelet-poor plasma was used in the present study to best reflect circulating 5-HT concentrations, and stabilizing agents were added to minimize inadvertent platelet activation, even though this risk has been estimated to be very low in horses.^35^^,^^66^ Comparison of results is further complicated by the use of different analytical techniques. In dogs, enzyme-linked immunosorbent assay systematically underestimates 5-HT concentrations compared with high-performance liquid chromatography, despite a good correlation between both methods.^10^ In horses, the use of liquid chromatography–tandem mass spectrometry to determine 5-HT concentrations in platelet-poor plasma has previously been validated.^67^ Median 5-HT concentrations in healthy horses were higher in Torfs et al. than in the present study, although the range of 5-HT concentrations was similar.^66^ In contrast, Delesalle et al. report a median 5-HT concentration in healthy horses comparable to that found in the present study, however, the observed range was substantially narrower, which might be attributable to the smaller sample size.^35^

The clinically irrelevant difference in NT-proBNP concentration between healthy horses and horses with MR observed in the present study contrasts with findings in humans and dogs, where increased NT-proBNP concentrations are associated with more advanced mitral valve disease.^20^^,^^22^^,^^23^^,^^68–71^ This might be due to the small number of horses with severe MR included in this study. In human and canine patients with mitral valve disease, NT-proBNP concentrations are positively correlated with echocardiographic indices of cardiac remodeling such as LA enlargement.^22–24^^,^^69^^,^^71^ In horses, only limited data regarding brain natriuretic peptide (BNP) and echocardiographic variables are available.^72^^,^^73^ Additionally, higher BNP concentrations are detected in horses with pulmonic valve regurgitation compared to healthy horses.^73^ However, BNP and NT-proBNP should not be considered equivalent biomarkers, as BNP has a shorter half-life and is therefore less stable than NT-proBNP.

Consistent with the present findings, Trachsel et al. reported no difference in cTnI concentrations between healthy horses and horses with MR.^74^ In contrast, Van Der Vekens et al. observed higher cTnI concentrations in horses with different types of valvular regurgitation and in horses with structural heart disease.^30^^,^^75^ As those studies included various cardiac disorders, whereas only horses with MR were included in the present study, the differing results might suggest that MR alone does not cause sufficient myocardial injury to increase cTnI concentrations. Additionally, while Van Der Vekens et al. used an Access Accu assay for cTnI analysis, we used a chemiluminescence immunoassay. Although both assays are validated for use in horses,^75^^,^^76^ the chemiluminescence immunoassay had a lower limit of detection (0.01 ng/mL) compared to the Access Accu assay (0.03 ng/mL).

When interpreting cTnI concentrations, the possibility of false-positive results should be considered. Of the 15 horses with an elevated cTnI concentration, only four also showed increased concentrations with additional cTnI and cTnT testing. This suggests that cTnI concentrations might have been falsely elevated in the remaining 11 horses. Multiple analytical and non-analytical causes of cardiac troponin elevation have been described, including septic shock, strenuous exercise, fibrin clot formation, and heterophile antibodies.^77^

In human and canine cardiology, multiple studies have described the prognostic value of cTnI and NT-proBNP as predictors of outcome. Although prognostic data on cTnI in MR are limited, higher concentrations are associated with adverse outcomes after mitral valve interventions in both humans and dogs.^26^^,^^78^^,^^79^ Even though cTnI is an established marker of myocardial injury in horses, its prognostic value in MR and cardiac disease remains limited.^74^^,^^80^ However, cTnI could serve as a prognostic biomarker in other equine diseases, including strangles and colic.^81^^,^^82^ In both human and canine mitral valve disease, NT-proBNP has demonstrated prognostic utility with defined cut-off concentrations associated with disease progression and survival.^70^^,^^71^^,^^83^^,^^84^ To the author’s knowledge, NT-proBNP has not been reported as a prognostic marker in horses.^28^ Combined cTnI and NT-proBNP measurement has demonstrated superior prognostic utility compared with either biomarker alone in humans and dogs.^27^^,^^85^ The current study did not allow assessment of the prognostic value of cTnI and NT-proBNP in horses with MR, as no follow-up data were available. The NT-proBNP concentrations were significantly higher in batch four and five, which could not be explained by unequal distribution of horses without and with MR, different sample storage time or inter-operator variability. Differences between batches would complicate individual horse monitoring over time. Therefore, the quality of the NT-proBNP ELISA test should be assessed in more detail before suggesting NT-proBNP could be used for serial monitoring in equine clinical patients.

### Study limitations

In some horses, concurrent valvular regurgitations were present in addition to MR. Even though MR was the predominant valvular regurgitation, this might have influenced the results. Additionally, the small number of severe MR cases might have prevented detection of significant biomarker differences. In some samples, storage time exceeded the predetermined maximum of 12 months which might have influenced the biomarker results. However, based on the available literature this negative effect was estimated to be rather limited.^86–89^ Since 5-HT and magnesium exhibit a circadian rhythm and the time of blood sampling was not standardized, this might have affected the results.^90^^,^^91^ When interpreting hypomagnesemia percentages, it should be considered that iMg concentrations were not corrected for potential changes in pH during sample handling and storage. However, in horses, the effect of pH on iMg is thought to be minimal.^17^ Additionally, sample handling was identical for all samples, meaning that any potential minor effects of pH on iMg concentrations would be consistent across all groups and data can thus still be compared. The measured billowing distance was a combination of actual MVB and mitral valve thickness, which slightly increases the measured MVB distance in horses with mitral valve thickening. In an attempt to define and diagnose MVP in horses, a horse-adapted cutoff value for the MVB distance is needed due to the different anatomy and size of the equine heart. Finally, except for LA area at end-systole, no additional echocardiographic measurements were included, as assessing potential correlations between biomarker concentrations and other echocardiographic variables (e.g., LV size or function) was beyond the scope of this study.

## Conclusions

In conclusion, the iMg concentration was significantly lower in horses with MR compared to horses without MR, but there was no significant association with MVB. No significant differences in 5-HT concentration were detected between healthy horses and horses with MR, which does not imply that 5-HT molecular mechanisms are not involved in the pathophysiology of equine MR. Additionally, an equine-specific MVB distance cutoff is required to distinguish excessive, abnormal MVB, better referred to as MVP, from normal, physiological MVB. No clinically relevant differences in NT-proBNP and cTnI concentration were found between healthy horses and horses with MR. The high biological variation in both NT-proBNP and cTnI potentially explains the lack of clinically relevant differences.

## Supplementary Material

Supplementary_material_aalag280

## Off-label antimicrobial declaration

Authors declare no off-label use of antimicrobials.

## Institutional animal care and use committee or other approval declaration

Approved by the Ethical Committee of the Faculty of Veterinary Medicine and Bioscience Engineering, Ghent University, Belgium (EC2023-09). Informed consent was obtained from the horse owners.

## Appendix

### Materials and methods

#### Animals and study design

Ethical approval for this prospective cross-sectional observational study was obtained by the Ethical Committee of the Faculty of Veterinary Medicine and Bioscience Engineering (EC2023-09). The study group consisted of horses with either no or trivial mitral valve regurgitation (MR) (healthy group) or mild, moderate, or severe MR (MR group). The majority of the sample population consisted of client-owned horses that were referred for cardiac examination while a limited number of horses were university-owned horses and horses donated for scientific research. All horses underwent a comprehensive cardiologic examination including auscultation, electrocardiographic recording at rest and during exercise, and echocardiography. The inclusion criteria were: Warmblood, Trotter, or Thoroughbred breed, ≥ 2 years of age, body weight between 400 and 700 kg, absence of congenital cardiac abnormalities, good-quality echocardiographic images, and no concurrent valvular regurgitation exceeding the severity of the MR classification.

#### Echocardiography

An echocardiographic examination with concurrent electrocardiogram recording was performed using Vivid iq or Vivid E95 ultrasound machine with M5Sc-RS or 4Vc phased array transducer (GE Healthcare, Diegem, Belgium). The exam contained two-dimensional, M-mode, color flow Doppler, and tissue Doppler images from a right and left parasternal view.^31^ The right parasternal four chamber view, the right parasternal short-axis view of the aorta and left atrium (LA) and right and left parasternal color flow Doppler images of the mitral valve were used for further analysis using EchoPAC software (Version 203, GE Healthcare, Diegem, Belgium).^31^

##### Mitral valve regurgitation classification

Mitral valve regurgitation was classified by a single observer according to a previously described MR scoring system developed by the Equine Department, University of Zürich (Switzerland) and the Equine Cardioteam Ghent, Ghent University (Belgium).^32^ The MR classification system consists of a four-fold assessment: (1) objective measurement of LA area, (2) subjective assessment of LA size and shape and (3) estimation of the regurgitant jet size and (4) jet duration (Supplementary item A). The LA area was measured from a right parasternal four chamber view at end-systole, one frame prior to mitral valve opening, and subsequently corrected to 500 kg using allometric scaling.^92^ The average of three measurements on three different cardiac cycles was used for MR classification. Cardiac cycles following a physiological or pathological arrhythmia were excluded for measurement. Subjective assessment of LA size and shape was performed based on a combined assessment of the right parasternal long- and short-axis view of the LA. Regurgitant jet size was estimated according to the proportion of the LA area occupied by the regurgitant jet on color flow Doppler images from a right and left parasternal long-axis view of the mitral valve. For jet duration it was assessed whether the jet was < 1/5 of LA area during the first half of systole, but ≥1/5 of LA area in the second half of systole. If a crescendo jet was present, the final score was subtracted by one as the jet was assessed to have a smaller hemodynamic impact. To avoid artificial creation of a crescendo jet due to a change in jet direction or through-plane motion, the MR jet was recorded and evaluated in multiple image planes. Currently, this MR scoring system has not yet been validated in horses against a gold standard or outcome variable. Nevertheless, by taking several factors into account that are relevant for determining MR severity, this classification system provides a standardized protocol for MR classification enabling reliable comparison of results from different research centers.

##### Mitral valve billowing assessment

Mitral valve billowing was defined as the degree of systolic displacement of one or both mitral valve leaflets above the mitral annular plane on a right parasternal four chamber view. The MVB distance was quantified by a single observer who was blinded for MR severity and MR jet duration, at end-systole, one frame prior to mitral valve opening, on a right parasternal four chamber view. First, the mitral annulus diameter was measured by connecting the ventricular side of the mitral annular hinge points. The mitral annulus diameter was normalized to a body weight of 500 kg using allometric scaling.^92^ Next, billowing of the septal and parietal mitral valve leaflet into the LA was quantified by measuring the billowing distance from the ventricular side of the mitral annulus to the atrial side of the most billowing portion of the septal and parietal mitral valve leaflet extending into the LA^33^^,^^34^ (Figure 1). The mitral annulus diameter and billowing distance were measured on three different cardiac cycles that were not preceded by a physiological or pathological arrhythmia. The average of the three measurements was used for statistical analysis.

### Sample collection and analysis

Venous blood was collected at rest before any exercise in two 30 mL syringes by puncturing the jugular vein with an 18G needle and carefully aspirating blood to avoid platelet activation. For all horses, blood was collected once and blood sampling was performed at different, random timepoints. Subsequently, the needle was removed from the syringe to gently transfer the blood to two cooled commercially available 9 mL ethylenediaminetetraacetic acid tubes (Vacuette, Greiner Bio-One, Kremsmünster, Austria), containing manually added stabilizing products (1 μmol/L clomipramine, 10 μmol/L phenelzine sulphate and 1 mmol/L acetyl salicylic acid),^35^ that were immediately stored on ice and used to obtain platelet-poor plasma. The remaining blood was transferred to three 8 mL serum tubes (Vacuette, Greiner Bio-One, Kremsmünster, Austria). Platelet-poor plasma for determination of serotonin (5-HT) concentration was obtained following a previously described protocol^6^ that limits the risk of inadvertent in vitro platelet activation, by a cooled double centrifugation step of the tubes (1st: 1500 g (2000 rpm), 15 min, 4 °C; 2nd: 2500 g (4000 rpm), 20 min, 4 °C) and consistent gentle handling of the samples. The obtained platelet-poor plasma was subsequently stored in cryovials at −80 °C and 5-HT concentrations were measured using liquid chromatography–tandem mass spectrometry (LC–MS/MS) (Xevo TQ-Absolute, Waters, Antwerp, Belgium). The use of this analytical technique has been previously validated in horses.^67^ Serum for analysis of ionized magnesium (iMg), cardiac troponin I (cTnI), and N-terminal pro-brain natriuretic peptide (NT-proBNP) was obtained by centrifuging the serum tubes within 30 min of collection at 2576 g for 10 min, and was stored in cryovials at −20 °C (cTnI) and −80 °C (iMg and NT-proBNP) for analysis. Ionized magnesium concentration was determined using the Stat Profile Prime VET ES (Nova Biomedical, Waltham, MA, USA), using an analytical method which has previously been used in horses to measure iMg.^55^ Cardiac troponin I concentration was determined by an external laboratory using a chemiluminescence immunoassay (ADVIA Centaur, Siemens, Forchheim, Germany) validated for horses.^76^ N-terminal pro-brain natriuretic peptide concentration was determined in-house using the Horse NT-proBNP ELISA kit (MBS014699, MyBioSource Inc., San Diego, California, USA). Samples were analyzed in a single measurement without replication and in several batches (5-HT: *n* = 5; iMg: *n* = 5; cTnI: *n* = 4; NT-proBNP: *n* = 5) with a maximum intended storage duration of 12 months. The limit of detection was 0.10 ng/mL for LC–MS/MS, 0.10 mmol/L for the Stat Profile Prime VET ES, 0.01 ng/mL for the chemiluminescence immunoassay and 31.2 pg/mL for the Horse NT-proBNP ELISA kit. Values below the detection limit were included for statistical analysis as 0.10 ng/mL for 5-HT, 0.10 mmol/L for iMg, 0.01 ng/mL for cTnI and 31.2 pg/mL for NT-proBNP. If cTnI concentration was > 0.06 ng/mL, one serum sample was re-analyzed for cTnI using the STAT high sensitive troponin-I reagent kit (Alinity, Abbott, Abbott Park, Green Oaks, Illinois, USA) and one serum sample was analyzed for cardiac troponin T using the Elecsys Troponin T high sensitive immunoassay (Cobas, Roche Diagnostics, Indianapolis, Indiana, USA) to check whether these were not falsely elevated.

### Statistical analysis

Data analysis was performed using SPSS Statistics 29 (IBM, Brussels, Belgium). Normality of the data was assessed by visual inspection of histograms and Q-Q plots and by performing the Shapiro–Wilk test. Normally distributed data are reported as mean ± standard deviation and non-normally distributed data are reported as median, interquartile range [Q1; Q3], and range (minimum-maximum). Homogeneity of variance was assessed using the Levene’s test. Demographic, echocardiographic, and biomarker data were compared between four groups (healthy, mild MR, moderate MR, and severe MR) and between two groups (healthy and MR). For normally distributed data, analysis of variance was used to compare four groups, and a *t-*test was applied for comparison between two groups. For non-normally distributed data, differences between four groups were assessed using the Kruskal–Wallis test, while comparison between two groups was made with the Mann–Whitney U test. Post-hoc Bonferroni correction was used for multiple comparisons. Multiple linear regression was performed to determine whether the presence or absence of MR and the different analysis batches had a significant effect on biomarker concentration. Spearman’s rho was used to assess correlations between biomarkers and echocardiographic data (cardiac dimensions and billowing distance). Spearman’s rho correlation coefficient (rs) was interpreted as follows: < 0.3 = poor; 0.3-0.5 = fair; 0.6-0.8 = moderate; > 0.8 = very strong.^93^ Additionally, Pearson chi-square was used to assess associations between MR severity and the presence or absence of hypomagnesemia. The strength of agreement was expressed using Cramer’s V: 0-0.05 = none; > 0.05-0.10 = weak; > 0.10-0.15 = moderate; > 0.15-0.25 = strong; > 0.25 = very strong.^94^ For every statistical test, *P* < 0.05 was considered statistically significant.

## Abbreviations

5-HT: 5-hydroxytryptamine or serotonin
BNP: brain natriuretic peptide
cTnI: cardiac troponin I
iMg: ionized magnesium
LA: left atrium/atrial
LV: left ventricle/ventricular
MMVD: myxomatous mitral valve disease
MR: mitral valve regurgitation
MVB: mitral valve billowing
MVP: mitral valve prolapse
NT-proBNP: N-terminal pro-brain natriuretic peptide
VICs: valve interstitial cells
