# Supplementary material for: Blood serotonin, ionized magnesium, cardiac troponin I, N-terminal pro-brain natriuretic peptide concentrations, and mitral valve billowing in horses with mitral valve regurgitation
Source: J Vet Intern Med. 2026 Nov 6;40(6):aalag280. doi: 10.1093/jvimsj/aalag280 (PMC13742131; doi:10.1093/jvimsj/aalag280)
Supplement: Supplementary file 1 [file supplementary_material_aalag280.zip › Supplementary_item_A_aalag280.docx]

1. LA area corrected to 500 kg using allometric scaling (adapted from Huesler and Schwarzwald (2016)^1^

| **R-LA area_s,end_ (500)** | **Points** |
| --- | --- |
| ≤ 95 cm^2^ | 1 |
| 96 cm^2^ – 110 cm^2^ | 2 |
| 111 cm^2^ – 125 cm^2^ | 3 |
| > 125 cm^2^ | 4 |

R-LA area_s, end_: left atrial area at end-systole, timed one frame prior to mitral valve opening on a right-sided parasternal long-axis view of the left atrium

2. Overall jet size assessment throughout systole on the R-LA & L-LA view

- Machine settings (adult horses): Probe frequency usually between 1.8 and 3.5 MHz, Pulse repetition frequency (PRF) / Nyquist limit (aliasing velocity [Va]) of ± 50-70 cm/sec (usually 1 step below maximum velocity range), high color gain that just eliminates random color speckles outside the blood pool^2^. Using a variance map, turbulent regurgitant blood flow is depicted by a green color^3^.
- Echocardiography: The echocardiographic exam should be performed at a physiological heart rate ranging between 28-48 bpm in absence of external stressors. The examination should be conducted in the unsedated horse. If sedation is required, the potential influence of sedatives on heart rate, preload, afterload and contractility, and therefore on LA size and function and on size of the regurgitant jet, need to be considered; in such cases, the scores will be of limited value and should be interpreted with caution.
- Scoring of jet size: The overall appearance of jet size throughout systole relative to the LA area is evaluated on several loops of the right (R-) and left (L-) parasternal LA view. Deviations from the standard long-axis LA plane are possible to maximize jet size. In case of different scores on the left and right side, the highest overall jet size score should be applied (adapted from Zoghbi (2017); Marr and Patteson (2010)^2,4^.

| **Cumulative jet size compared to LA area** | **Points** |
| --- | --- |
| - Very small jet, within < 1/5 of LA area | 0 |
| - Small jet, within ≤ 1/3 of LA area | 1 |
| - Moderate jet, within ≤ 1/2 of LA area | 2 |
| - Large jet, > 1/2 of LA area | 3 |
| **Additional consideration of jet duration** | **Points** |
| - Jet < 1/5 of LA area 1^st^ half of systole but ≥ 1/5 in 2^nd^ half of systole | -1 |

3. Subjective LA size and shape assessment on a right parasternal long-axis and short-axis view focused on the LA (combined assessment from both views).

| **Criteria evaluated** | **Points** |
| --- | --- |
| - Normal LA appearance, no enlargement - Normal LA shape | 1 |
| - Appearance of mild LA enlargement - Normal or roundish LA shape | 2 |
| - Appearance of moderate LA enlargement - Rounded LA shape | 3 |
| - Appearance of severe LA enlargement - Round LA shape | 4 |

4. Total score

| **Point Score** | **Severity** |
| --- | --- |
| 2 | Trivial |
| 3 - 5 | Mild |
| 6 - 8 | Moderate |
| 9 - 11 | Severe |

References

1. Huesler IM, Mitchell KJ, Schwarzwald CC. Echocardiographic Assessment of Left Atrial Size and Function in Warmblood Horses: Reference Intervals, Allometric Scaling, and Agreement of Different Echocardiographic Variables. J Vet Intern Med 2016;30:1241-1252.

2. Zoghbi WA, Adams D, Bonow RO, et al. Recommendations for Noninvasive Evaluation of Native Valvular Regurgitation: A Report from the American Society of Echocardiography Developed in Collaboration with the Society for Cardiovascular Magnetic Resonance. J Am Soc Echocardiogr 2017;30:303-371.

3. Blissitt KJ, Bonagura JD. Colour flow Doppler echocardiography in horses with cardiac murmurs. Equine Vet J Suppl 1995:82-85.

4. Marr CM, Patteson M. Chapter 9 - Echocardiography. In: Marr CM, Bowen IM, eds. Cardiology of the Horse (Second Edition). Edinburgh: W.B. Saunders; 2010:105-126.
